# Evaluating the longitudinal effectiveness of a smoking cessation counselling course based on the 5A model for medical students in family medicine placement

**DOI:** 10.3205/zma001734

**Published:** 2025-02-17

**Authors:** Jessica Ruck, Elena Tiedemann, Jessica Sudmann, Andrea Kübler, Anne Simmenroth

**Affiliations:** 1 University Hospital Würzburg (UKW), Department of General Practice, Würzburg, Germany; 2 University of Würzburg, Department of Psychology I, Würzburg, Germany

**Keywords:** medical teaching, smoking cessation, counselling, prevention, communication skills, 5A model

## Abstract

**Background:**

Preparing students for their future roles in preventive medicine is relevant, especially on the subject of nicotine consumption. We implemented a longitudinal smoking cessation counselling course as a part of the subject “prevention and health promotion”. Beginning with 6^th^ semester students were taught the 5A model, a widely used framework to support behavioural change. Four semesters later, we evaluated feasibility of the counselling in the family medicine placement.

**Methods:**

In this evaluative follow-up study, we used online questionnaires with Likert-scales, closed and open questions. Students of the 10^th^ semester assessed feasibility and obstacles of counselling during placement in a family practice with real patients, their counselling confidence and professional attitudes. For examination of changes since 6^th^ semester we compared matched data. Data were analysed with t-tests and ANOVAs.

**Results:**

Data of 114 students of the 10^th^ semester were analysed, 45 data could be matched to the 6^th^ semester. Results showed that the intervention was feasible under favourable conditions: half of the students did not perform a counselling because of lack of time or opportunity. Performing the counselling during the placement led to a greater increase in felt counselling competences compared to students who did not perform a counselling.

**Conclusion:**

To improve communicative skills in the area of behavioural change, it is important to combine theoretical content and practical implementation. The 5A model has proven due to its simplicity. Promoting good framework conditions in family medicine, such as sufficient time, is essential to give students the opportunity to practice counselling and increase their felt competence.

## Zusammenfassung

**Hintergrund:**

Die Vorbereitung von Studierenden auf ihre künftige Rolle in der Präventivmedizin ist von hoher Bedeutung, insbesondere im Hinblick auf den Nikotinkonsum bei Patient:innen. Wir haben einen Längsschnittkurs zur Raucherentwöhnungsberatung als Teil des Fachs „Prävention und Gesundheitsförderung“ eingeführt. Im 6. Semester wurde den Studierenden das 5A-Modell vermittelt, ein weit verbreitetes Rahmenkonzept zur Unterstützung von Verhaltensänderungen. Vier Semester später evaluierten wir die Anwendbarkeit der Beratung in der hausärztlichen Praxis im Blockpraktikum.

**Methoden:**

In dieser evaluativen Folgestudie verwendeten wir Online-Fragebögen mit Likert-Skalen, geschlossenen und offenen Fragen. Die Studierenden des 10. Semesters bewerteten die Durchführbarkeit und die Hindernisse der Beratung mit realen Patient:innen während des Blockpraktikums in einer Hausarztpraxis, ihre wahrgenommene Sicherheit und ihr Kompetenzerleben in der Beratung, und ihre Einstellungen zum Thema Rauchen. Zur Untersuchung der Veränderungen seit dem 6. Semester wurden die Daten miteinander verglichen. Die Daten wurden mit t-Tests und ANOVAs ausgewertet.

**Ergebnisse:**

Die Daten von 114 Studierenden des 10. Semesters wurden analysiert und 45 Datensätze konnten mit denen des 6. Semesters verglichen werden. Die Ergebnisse zeigten, dass eine Beratung unter günstigen Bedingungen durchführbar war: Die Hälfte der Studierenden führte aufgrund fehlender Zeit oder Gelegenheit keine Beratung durch. Die Durchführung der Beratung während des Praktikums führte zu einem größeren Anstieg der subjektiv wahrgenommenen Beratungskompetenz im Vergleich zu Studierenden, die keine Beratung durchführten.

**Schlussfolgerung:**

Um die kommunikativen Fähigkeiten im Bereich der Beratung bei Verhaltensänderungen zu verbessern, ist es wichtig, theoretische Inhalte und praktische Umsetzung zu verbinden. Das 5A-Modell hat sich aufgrund seiner Einfachheit bewährt. Die Förderung guter Rahmenbedingungen in den Hausarztpraxen, wie z.B. ausreichend Zeit, ist essentiell, um den Studierenden die Möglichkeit zu geben, Beratung zu üben und ihr Kompetenzerleben zu erhöhen.

## 1. Background

Tobacco smoking is considered to be a risk factor for many oncological, cardiovascular and respiratory diseases and the leading cause of premature death [1], [2]. Every year, 6 million deaths worldwide and 125 000 deaths in Germany are related to tobacco use [3], [4]. With annual costs of €97.24 billion (31% direct and 69% indirect costs) smoking has also an immense impact on the German economy and the health care system [5]. Even though quitting tobacco smoking has substantial and long term health benefits for smokers of all ages [6], more than 30% of Germans still smoke [7].

Without professional support 95-97% of smoking cessations fail [8], while medical assisted smoking cessation through brief counselling and medical advice is more effective [9], [10]. Evidence-based behavioural support (e.g. brief counselling by medical staff) can significantly increase abstinence rates [11], [12]. Although effective interventions are available, only a few of the smokers in Germany receive guideline-based treatment for their tobacco dependence [10]. The German DEBRA-study showed that 72.6% of smokers were not asked about their smoking behaviour by their general practitioner (GP) and just 14.4% of smokers were advised to stop smoking. Only a small percentage of GPs recommended medical or psychological treatments [11]. Brief smoking cessation interventions are still too rarely implemented in primary care. The reasons given by GPs are structural obstacles like time and reimbursement of costs but also a lack of training in smoking cessation counselling and missing knowledge about cessation methods [13], [14], [15], [16]. This could be due to the insufficient presence of the topic smoking cessation in teaching and due to a strong industrial lobby favoring nicotine consumption. Results of the Global Health Professions Student Survey with 31 countries showed that less than 40% of medical students named smoking cessation counselling as a part of their curriculum [17]. In a German study, less than 3% of medical students reported about performing practical exercises and four out of five German medical students at the end of their studies did not know how to treat a smoker regarding to a cessation [18].

Preparing students for their future roles in preventive medicine is relevant [19] and smoking cessation training programs can improve knowledge as well as attitudes and skills of smoking cessation counselling [20]. Ambulatory placements can be seen as a good setting for teaching students to practice smoking cessation counselling and to increase the respective competence [19].

**Figure 1:**
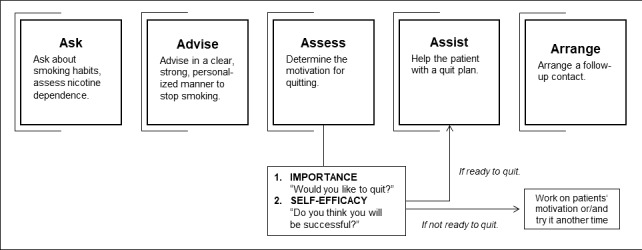
The 5A model (figure created by the authors based on WHO 2014 [35])

Addressing this teaching gap, the Department of General Practice at the University Hospital Würzburg (UKW) has implemented a course of smoking cessation counselling on the basis of the 5A model in the winter semester 2018/19, which has been part of an existing cross-sectional teaching subject on “prevention and health promotion” [21]. The 5A model (ask, advise, assess, assist, arrange; see figure 1 (Fig. 1)) is a valid and widely used brief verbal intervention for smoking cessation that is based on Motivational Interviewing according to W. R. Miller and S. Rollnick [22], [23], [24]. The approach is recommended in national and international guidelines and is used for supporting patients in behavioural change (e. g. quit attempts and smoking cessation) [22], [23]. Following G. E. Miller’s learning pyramid [25], factual knowledge, practical knowledge and practical communication skills were integrated and practiced within a protected context with peers and later with real patients in an ambulatory setting. This follow-up study evaluated the implemented smoking cessation counselling course regarding to the attitudes towards smoking cessation and the feasibility of the learned 5A model in practice, and the acquired self-confidence in counselling (see figure 1 (Fig. 1)). Therefore, the same cohort from 2018/19 was investigated four semesters later in the winter semester 2020/21, after students had a family medicine placement in family practices.

We assumed that

- putting the learned smoking cessation counselling into practice would be feasible,
- performing a counselling in the placement would influence confidence and attitudes towards smoking cessation counselling in a positive way,
- students show an increase in learning regarding to smoking cessation counselling and its application in practice.

We were also interested in practical problems of performing a smoking cessation counselling during placement.

## 2. Methods

### 2.1. Samples and study design

**Figure 2:**
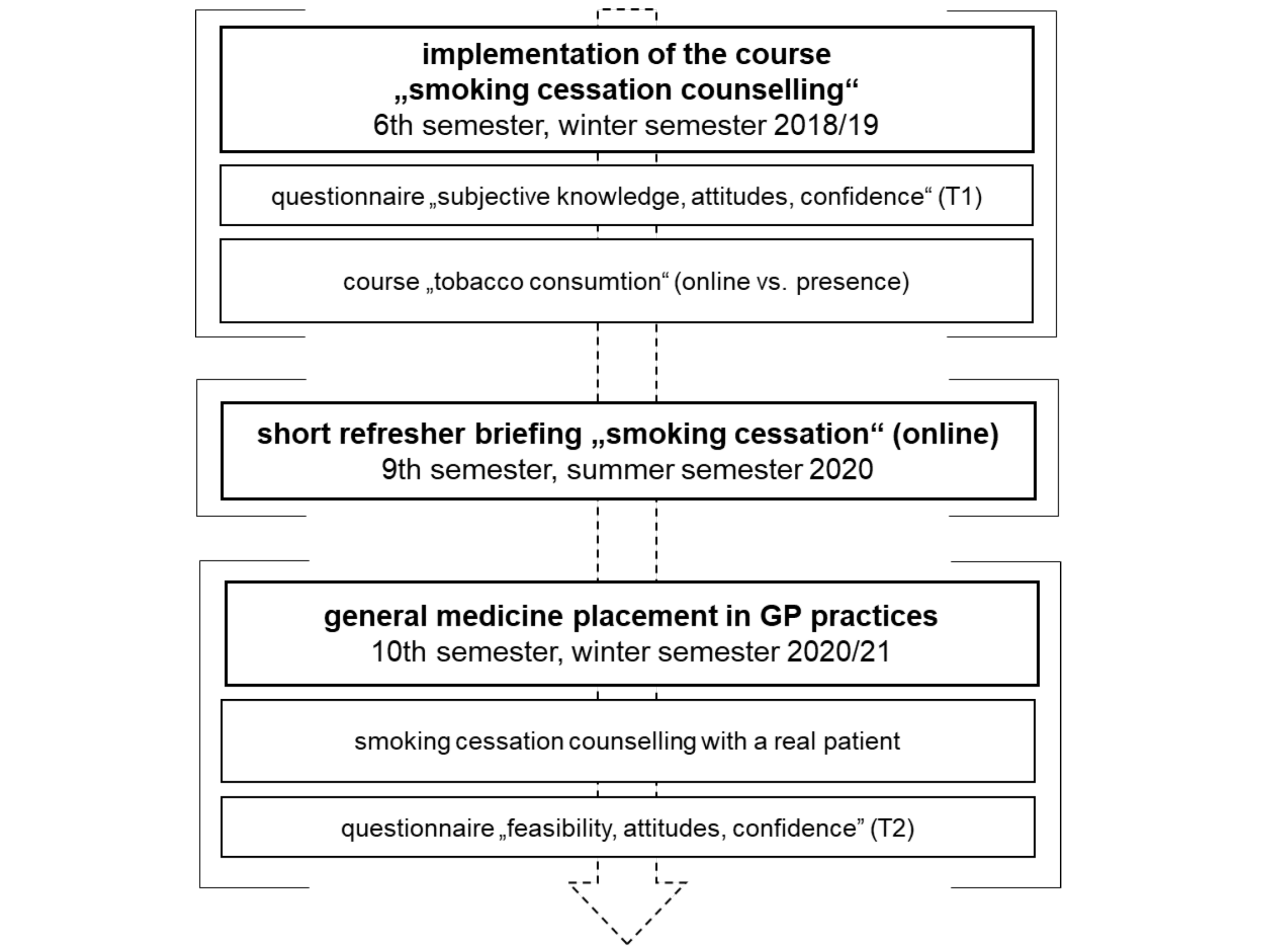
Study design

This evaluation study is a follow-up of a previous study published elsewhere [21]. Data from this previous study (T1) were used for a comparison with data after the placement in family medicine (T2) to analyse changes over time. Figure 2 (Fig. 2) illustrates the study design followed by a description below.

#### 2.1.1. Winter semester 2018/19 (6^th^ semester)

In a previous study in the winter semester 2018/19 (T1) we evaluated the didactic methods of the new course. Medical students of the 6^th^ semester participated either in a presence course or in an online training. The course (90 minutes) included theoretical basics on smoking and smoking cessation, an introduction to the 5A counselling concept, and either teaching videos of a consultation (online teaching) or practice sessions in small groups (presence). As a baseline, an online self-assessment questionnaire about individual knowledge about smoking and attitudes towards smoking cessation was filled in before attending the course (T1). From 145 participants, 130 data sets could be analysed [21].

#### 2.1.2. Summer semester 2020 (9^th^ semester)

During the 9^th^ semester, a short refresher on the content of the smoking cessation course of the 6^th^ semester was given was given to the same students using a PowerPoint presentation.

#### 2.1.3. Winter semester 2020/21 (10^th^ semester)

Four semesters after the first training, the same cohort (now 10^th^ semester) was examined again (154 potential students). These students were enrolled in a placement in family medicine. The placement is an obligatory two-week practical training in an ambulatory setting of a family practice. Students accompany a GP the whole day and independently assess medical histories, perform physical examination and diagnostics, supervised by GPs. During these two weeks, students should be given the opportunity to conduct at least one smoking counselling session with a smoking patient. GPs did not receive a smoking cessation training, but were asked to search for suitable patients (e. g. with initial medical history or patients coming for a check-up). Afterwards, students filled in an online self-assessment questionnaire (T2) about counselling feasibility towards the 5A-model, self-confidence and attitudes about smoking cessation counselling. Students were also asked for feedback about the new course of smoking cessation. With an individual, pseudonymised code, the questionnaires from the 6^th^ and 10^th^ semesters could be matched.

#### 2.1.4 Study sample

**Figure 3:**
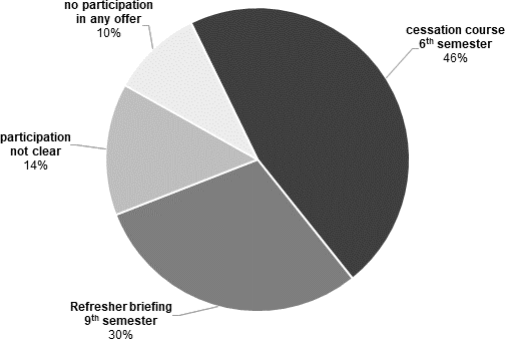
Percentage of students (n=114) in 10^th^ semester who participated in another course offering

Data of 114 students were analysed. Of these 114 students (see figure 3 (Fig. 3)), 53 students had participated in the cessation course in the 6th semester and 34 had participated in the refresher in the 9^th^ semester. For 16 students the participation was unclear and eleven did not participate in any teaching offer.

### 2.2. Measuring material

#### 2.2.1 Self-assessment questionnaires in the 6^th^ semester (winter semester 2018/19)

The questionnaire (see attachment 1 , supplement material 1) used closed items with a five-point Likert scale (1=*strongly disagree*, 5=*strongly agree*), single and multiple choice questions and open questions. Students had been asked about sociodemographic data, individual smoking status, and attitudes toward smoking, self-assessment of knowledge about smoking, self-assessment of counselling skills, and past practical experiences.

#### 2.2.2. Online self-assessment questionnaire in the 10^th^ semester (winter semester 2020/21)

The online questionnaire (see attachment 1 , supplement material 2) comprised 38 items with closed items using again a five-point Likert scale (1=*strongly disagree*, 5=*strongly agree*), single and multiple choice questions and open questions. Students were asked about sociodemographic data, individual smoking status, personal assessment of the course in school grades (1=*very good*, 6=*insufficient*), feasibility of counselling, attitudes toward smoking and self-assessment of self-confidence and counselling skills. For measuring changes over time, three items of the questionnaire were taken from the questionnaire presented in the 6^th^ semester. The questionnaires were developed and provided online with the online tool EvaSys^®^.

### 2.3. Statistical analysis

Data cleaning reduced the number of 116 filled questionnaires in the 10^th^ term to 114: In case of one identical code in two questionnaires, only the latter was considered. One participant withdrew from participation. Through pseudonymized codes, data sets from the 6^th^ and the 10^th^ semester could be matched provided the students remembered their individual code correctly. Of the original cohort of N=130, n=45 students (35%) were included for a comparison over time. Categorial data were shown as frequencies and interval-scaled data such as mean, and standard deviation. To check for abnormal values, z-standardization [26] was performed. To check the randomness of missing values, the MCAR (Missing completely at random) test according to Little [27] was performed. Group differences in sociodemographic items for categorical data were tested with the chi-square test (χ^2^), and continuous data such as age with Welch's t-test or one-way Welch’s ANOVA. In the case of two independent samples, following the recommendation of Rasch et al., we omitted pre-testing of the assumptions and applied the Welch-test, which is less error-prone than the two-sample t-test [28]. To analyse the feasibility of a smoking cessation counselling on a real patient, frequencies of conduction (yes/no) were calculated. In addition, two new variables were formed, “confidence” (4 items: Confidence 1-4) and “attitude” (4 items: attitude 1-4) (see attachment 1 , supplement material 3). The scales were formed using a mean value calculation; cases with missing values on individual items were excluded for the respective scale. For tests of group differences, Welch’s t test was chosen for interval-scaled variables. As the categories assessed different levels, Holm–Bonferroni method for multiple comparisons was applied separately for each category [29]. To analyse changes over time, t-tests for dependent samples were calculated. SPSS 26.0 was used to conduct statistical analyses. The significance level was set to α<.05.

### 2.4. Data management, data protection and ethics

Study information was given in the family medicine courses (9^th^ semester) and via e-mailing lists. Further information, consent declarations and the online study link were available on the e-learning course of the University. Vouchers were raffled among participants. All participants and teaching GPs were informed about the study procedure, data privacy and contact data to ask questions. The data were collected via questionnaires in pseudonymized form using a code with 6 digits created by the participants to allow for proper matching of subsequent data. Participants signed informed consent and agreed to processing their personal data for the purpose of the study. Data was stored electronically only on password-protected media of the Department of General Practice of the University of Würzburg and deleted if participation was withdrawn. The Ethical Review Board of the Medical Faculty of University of Würzburg approved the study in March 2020: identification number: 20200302 02).

## 3. Results

### 3.1. Feedback of students

After the placement, the course program received the German school overall grade 2.5 (SD=0.8; 1=*very good*, 6=*insufficient*) by the students. In open-ended questions, students confirmed that implementation of smoking cessation training was reasonable. They wished for more practical exercises and more information about nicotine substitute products.

### 3.2. Demographic characteristics of students (10^th^ semester, placement)

Of the included 114 students, 69.3% were female with a mean age of 26 years. More details about demographic data, smoking status and previous medical apprenticeship before studying are shown in table 1 (Tab. 1).

### 3.3. Feasibility of consultation in practice

#### 3.3.1. Feasibility of consultation

Half of the students (n=57) conducted a smoking cessation counselling on a real patient during their placement (counselling yes, CY) while 57 students did not (counselling no, CN, n=57). There were no group differences between the semesters regarding to sociodemographic, smoking status or previous apprenticeship. The groups CY and CN only differed in their previous medical apprenticeship before studying.

#### 3.3.2. Duration of counselling and counselling context

For most CY-students, the time required (see table 2a (Tab. 2)) was between 5 and 10 minutes and the time required was considered feasible (M=4.1, SD=0.9). Most counselling was performed in the context of history taking (see table 2b (Tab. 2)). Obstacles for counselling reported by CN-students are shown in table 2c (Tab. 2). CN-students indicated significantly less support from their teaching GPs in finding a suitable patient than CY students (*t*(106.3)=2.1, *p*=0.043).

### 3.4. Confidence in counselling

#### 3.4.1. Practicing the 5A model

Of CY-students, 66.7% (n=38) have completely and 33.3% (n=19) have partly performed their consultation based on the principles of the 5A model. Students used the first two steps “ask” and “advise”. The other “As” of the model were used at least partially (see table 3 (Tab. 3)).

#### 3.4.2. Confidence in using the 5A model

On average, CY-students felt confident in their counselling performance (M=3.9, SD=0.8) and were satisfied with it (M=3.9, SD=0.9) *(“overall, I felt confident in conducting the brief intervention based on the 5A model.”*). Table 3 (Tab. 3) shows confidence in practicing each “A” of the model (*“I felt confident in the use of the ask”/“advise”/“assess”/“assist”/“arrange” item.”*).

### 3.5. Comparison between CY and CN regarding attitudes, confidence and competences

#### 3.5.1. Attitudes and confidence in counselling

Students of CY- and CN-group differed significantly in their average attitude and counselling confidence (see table 4 (Tab. 4)).

#### 3.5.2. Change in competences

After the placement, 72% of CY-students (n=41) reported that competences in smoking cessation counselling had increased while 86% of CN-students reported no changes (n=49). This change in counselling skills reported from CY-students after the placement was significantly increased as compared to CN-students (*t*(79.2)=8.2, *p*<0.001, see table 4 (Tab. 4)).

#### 3.6 Semester comparison over time (6^th^ vs. 10^th^ semester)

For 45 students of the 10^th^ semester, complete questionnaire data sets from the previous study 2018/19 (T1) and the placement 2020/21 (T2) were available. For a comparison over time, the items “attitude 1”, “attitude 2” and “confidence 1” were included, all comparisons were statistically significant.

#### 3.6.1. Attitudes T1 vs. T2

**Figure 4:**
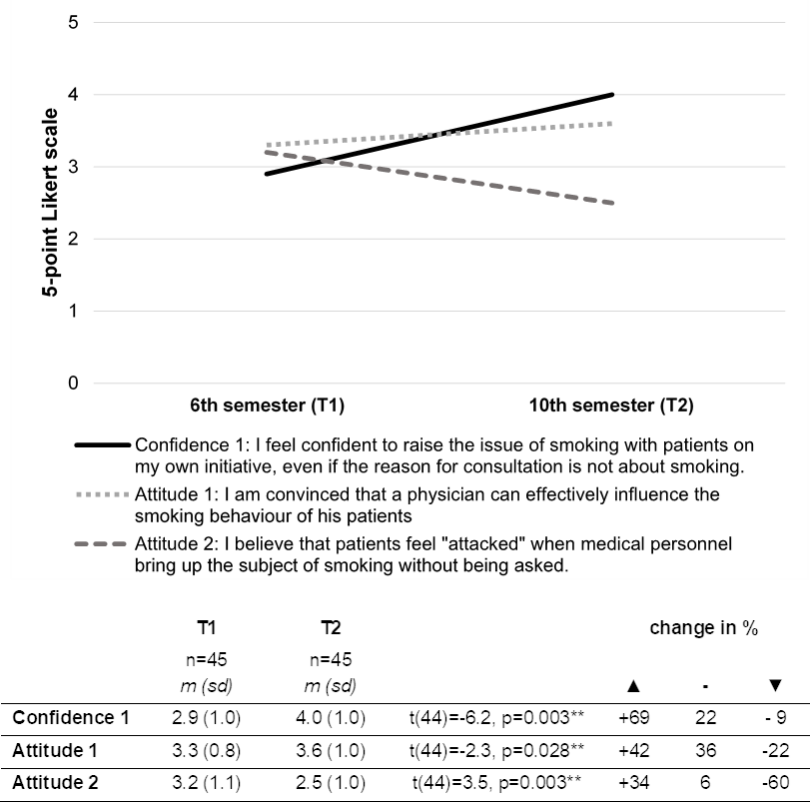
Changes of self-assessment over time for items “confidence 1”, “attitude 1” and “attitude 2”; using a 5-point Likert scale (1=strongly disagree, 5=strongly agree)

Forty-two percent of the students were more convinced that they could effectively influence the smoking behaviour of patients (“attitude 1”; see figure 4 (Fig. 4)). The worries that patients could feel attacked by the counselling decreased about 60% (“attitude 2”; see figure 4 (Fig. 4)).

#### 3.6.2. Confidence in counselling before and after (T1 vs. T2)

The confidence of students to address the issue of smoking significantly increased about 69% over time (“confidence 1”; see figure 4 (Fig. 4)).

## 4. Discussion

This follow-up study evaluated the practical feasibility of the 5A model learned in the newly implemented smoking cessation course, which students attended during T1, and the changes in students’ attitudes and confidence regarding counselling. Results showed that the short-intervention was feasible under favourable conditions, i.e. support by the GP. The longitudinal course influenced attitudes in a positive way and increased counselling confidence of students. The students who performed the short-intervention in the placement (CY) showed a greater increase in their confidence in counselling skills than students who did not perform (CN).

These results are in line with other evaluations of smoking cessation courses showing improvement in confidence and attitudes [19], [20], [30], [31]. Leong et al. (2008) for example, evaluated that performing a smoking cessation in a placement led to positive changes in students’ attitudes and knowledge [20]. Half of the students did not perform a consultation (CN) because of reported unfavourable conditions such as “lack of time or lack of opportunity”. We do not know exactly, what students have subsumed under this item, perhaps also factors on the part of the attending physicians. As shown in studies with GPs, lack of time is a common obstacle in practice [13], [14], [15], [16]. However, the fact that the other half of the students (CY) were able to do some counselling in an adequate time raises the question how the general placement conditions of the students had influenced the feasibility of counselling. CN-students reported less support from their teaching GPs in finding a suitable patient. Possible reasons could be external obstacles, attitudes of the GPs towards smoking cessation counselling or a reciprocal effect with the attitudes of CN-students.

The German SNICAS-study describes that internal reasons like individual attitudes of GPs influence decisions of indication and intervention towards smoking cessation counselling [32], [33]. Although GPs are aware of the relevance of smoking cessation, they rarely perform a counselling. Even if 80% used any intervention, most of them used medications or nicotine replacement products. Only 6% conduced counselling and only 11% used the ideal standard of the combination of all these three interventions [33]. The authors regarded the pessimistic attitudes toward feasibility and effectivity of short-interventions as a possible explanation [32]. As attitudes can influence behaviour, further studies should focus on the role of teaching GPs as well as on their own smoking status. Maybe a more positive attitude of teaching GPs or an explicit request for helping students could improve the support of teaching GPs in finding a suitable patient for counselling. GPs as role models and good clinical teachers are important especially in the teaching of soft skills as good communication or counselling.

Attitudes of the students could have had also influenced their behaviour. Although there were no differences in particular items of attitudes, CN-students had a more negative overall attitude toward smoking cessation counselling as compared to CY-students. Pessimistic attitudes may have prevented CN-students from engaging in counselling. Since just the overall attitude was significantly different and the attitudes were only asked after the placement, it cannot be ruled out that the CN-groups attitude worsened retrospectively, since they did not carry out any counselling. After participation of the new smoking cessation course, students felt more confident in counselling.

Our results show that performing a smoking cessation in practice with a real patient is a worthwhile part of the course. This can be seen in the significant increase of counselling competences in CY-group as compared to CN-group. As already reported, a common obstacle for GPs to perform a smoking cessation counselling is a lack of training and time. GPs ascribe great importance to consulting competences [32] and adequate training seems to be a key factor to enhance engagement of GPs’ smoking cessation counselling [34]. Our results confirm that smoking cessation counselling training of students with a practical focus is necessary to strengthen competences of future physicians [30].

### Limitations

Firstly, a structural, objective measurement of competences e.g., with Objective Structured Clinical Examination (OSCE) is missing. An OSCE could be a useful supplement to the subjective assessment of the perceived competence. Due to the high organizational and financial costs of an OSCE, this has not been applied. Secondly, the comparison among the students is limited because the placements of students were in various practices of family medicine differing in location, size and supervision. Thirdly, not all students of the 10^th^ semester underwent the smoking cessation course in the 6^th^ semester. Thus, the knowledge of the students differed at the beginning, which limits comparability. A short introduction in smoking cessation counselling for all students before the placement would have been a better starting point, but could not be realized for practical reasons.

## 5. Conclusion

The newly implemented course for smoking cessation counselling has closed an important gap in the medical curriculum of the University of Würzburg. Since 2022, every student has to participate in the course in the 6^th^ semester and has to perform a brief counselling with a smoking patient during their family medicine placement at the end of their 10^th^ semester. Our results show the practical feasibility of a stepwise 5A consultation and respective counselling, and an improvement of students’ competences and professional attitudes, especially through practice with a real patient. As there seems to be evidence that the attitude of teaching GPs towards smoking cessation counselling influences their readiness to teach it to students, future studies should investigate how to promote supportive conditions. Additionally, teaching GPs should be better trained to support students when it comes to smoking cessation counselling.

## 6. Practice implications

Still too many people die as a result of the preventable health consequences of smoking. For this reason, smoking cessation counselling should play an important role in family medicine and most clinical disciplines, but is applied unfortunately far too little. Considering that even a short smoking cessation counselling is effective, smoking cessation is still given far too little attention in the medical curriculum as a whole. Smoking cessation modules such as presented here are easy to implement and indispensable to train future doctors to increase frequency and quality of their respective counselling.

## Authors’ ORCIDs

- Jessica Ruck: [0009-0001-2931-8157]
- Elena Tiedemann: [0009-0001-2931-8157]
- Andrea Kübler: [0000-0003-4876-0415]

## Competing interests

The authors declare that they have no competing interests. 

## Supplementary Material

Supplements

## Figures and Tables

**Table 1:**
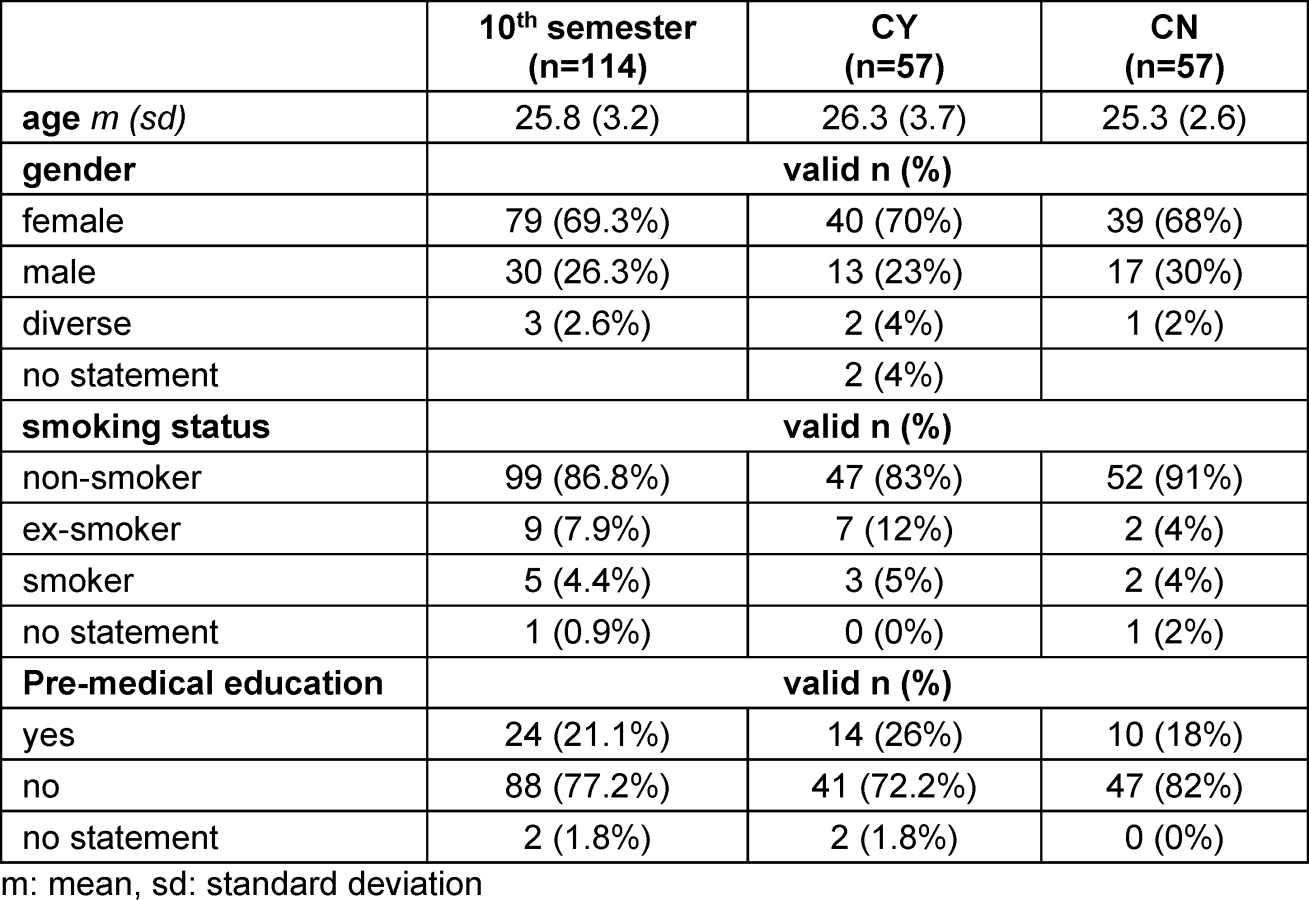
Demographics, smoking characteristics and previous medical apprenticeship for all students of 10^th^ semester and separated into counselling groups (CY: counselling yes, CN: counselling no)

**Table 2:**
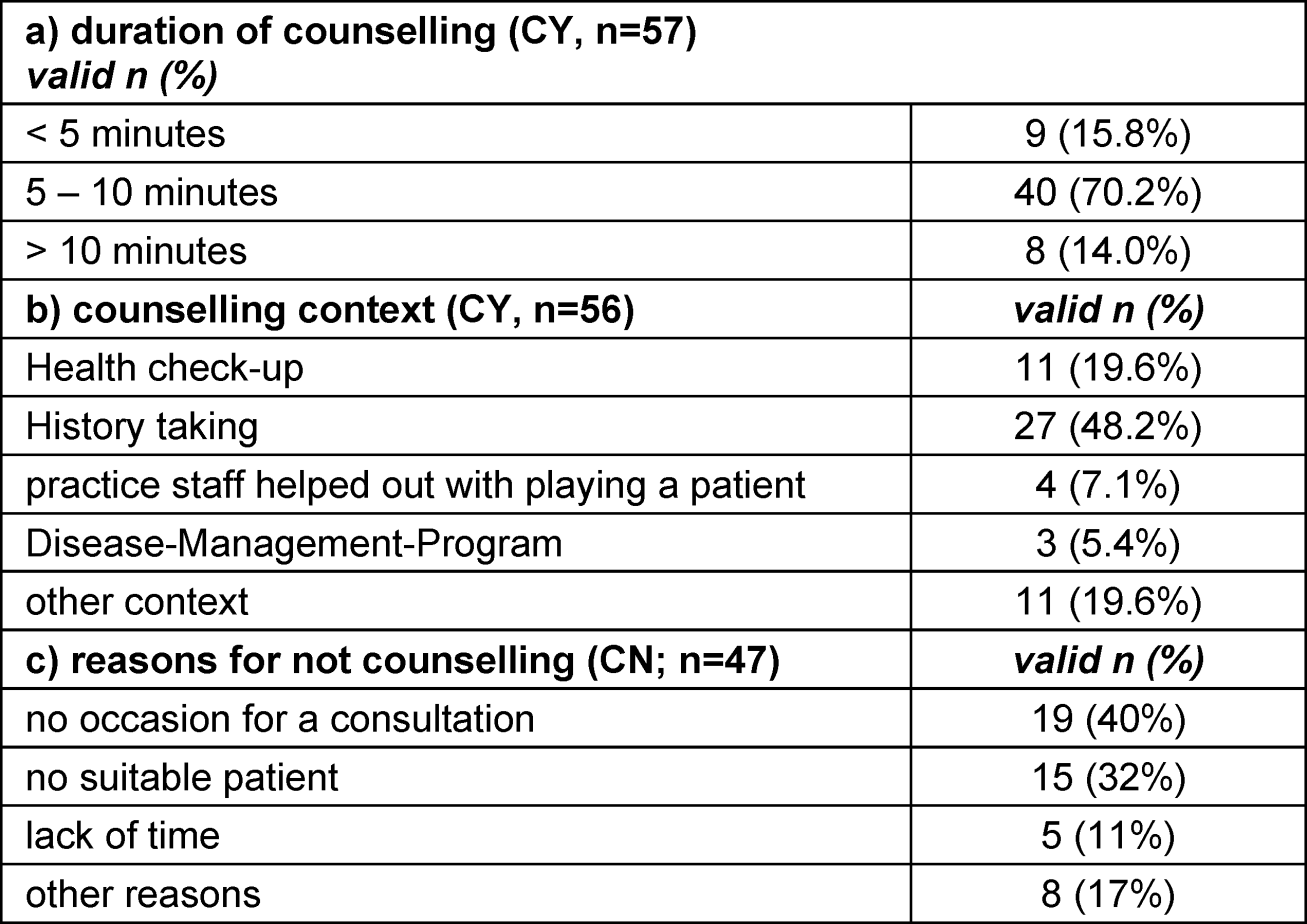
Feasibility of counselling: duration, context and problems

**Table 3:**
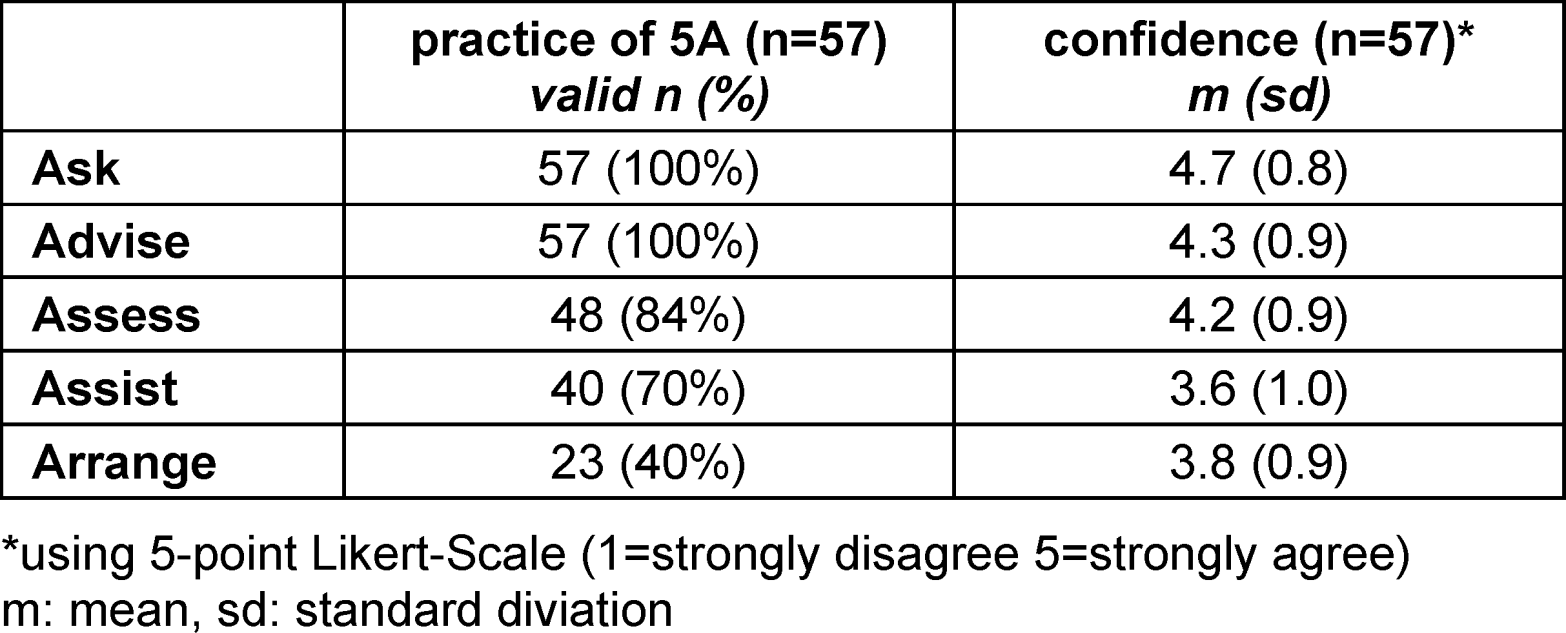
Practice and confidence for each “A” of the 5A model (CY-students)

**Table 4:**
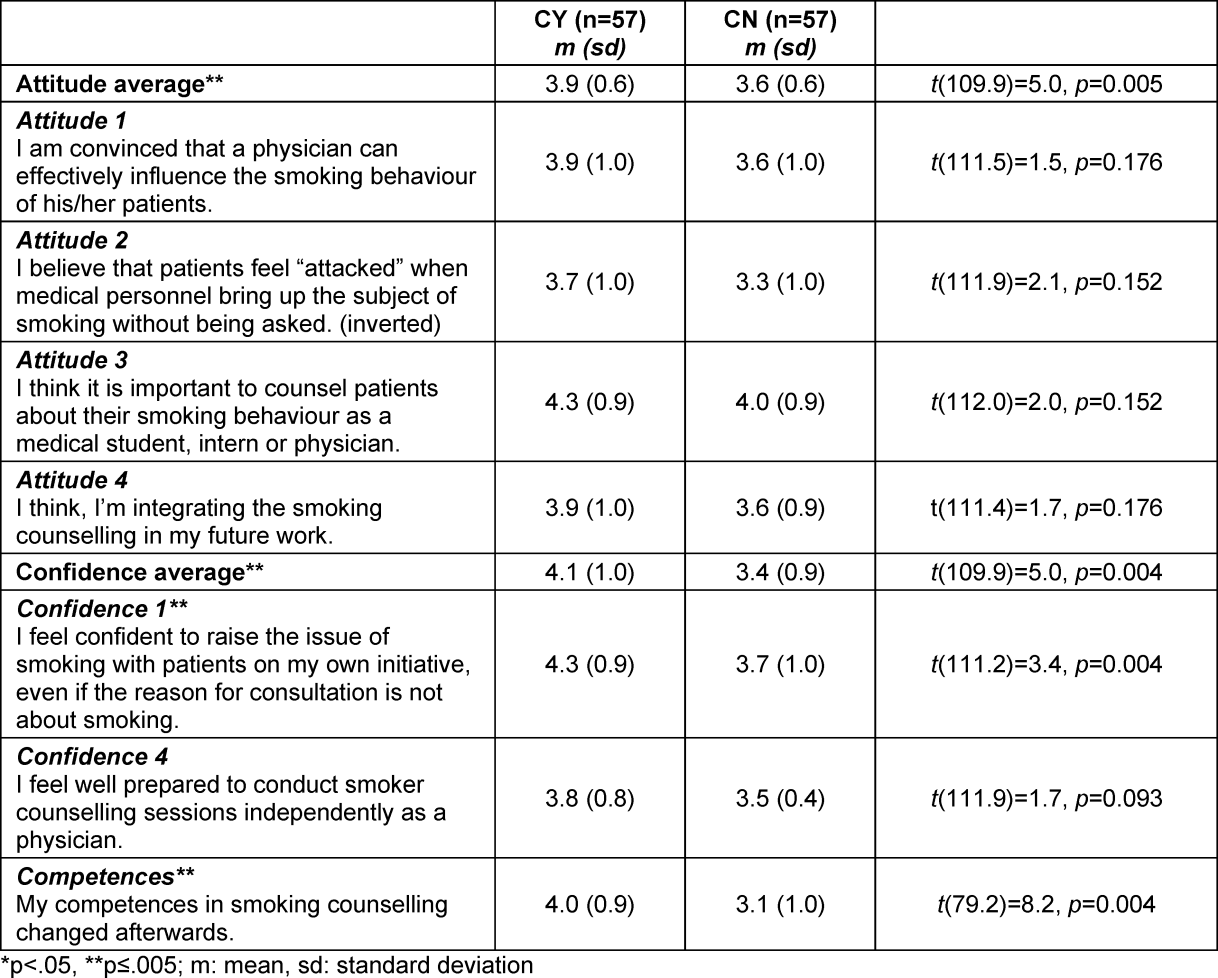
Comparison between CY and CN; using 5-point Likert scale (1=strongly disagree, 5=strongly agree)
