# Supplementary material for: Evaluating the longitudinal effectiveness of a smoking cessation counselling course based on the 5A model for medical students in family medicine placement
Source: GMS J Med Educ. 2025 Feb 17;42(1):Doc10. doi: 10.3205/zma001734 (PMC12086244; doi:10.3205/zma001734)
Supplement: Supplements [file JME-42-10-s-001.pdf]

Attachment 1: Supplements

Supplemental material 1: Self-assessment questionnaires in 6th semester (winter semester 2018/19)

1. Information about the course and data management

Tobacco smoking is the most important preventable cause of death worldwide and also in Germany. While the topic comes up repeatedly in various courses, ways to treat patients who smoke are rarely addressed specifically. This winter semester, there will be a separate course on the theme of prevention for the first time. After a short theoretical part, the focus of the event will be on training your practical skills to conduct a short consultation with a patient who smokes. Two different teaching methods will be used to teach this content: One is e-learning in an an online course, and on the other hand a present seminar. The learning objectives are identical in both courses. Your semester has been randomized into two groups, you will find your assignment (either online course or seminar) in the course room on wuecampus. In the exam in the subject Prevention you will probably be asked three questions on the topic of will be asked. At the end of the semester, 20 students from each group will be asked to participate in an OSCE. The randomly selected will be informed by mail and will conduct a counseling interview with standardized patients. The OSCE is voluntary, completely independent of the written exam, and does not affect its grading. We will critically follow the introduction of the new course and compare the different teaching methods compare the different teaching methods. In addition to participating in the OSCE, we ask you to fill out two questionnaires: the first today and the second immediately after the course. By answering the questions and giving your feedback, we will be able to we will be able to evaluate the best teaching methods and improve the courses for the coming semesters on this important topic.

Thank you for your assistance!

Note on data managment
The data collected (answers from questionnaires, results of individual questions from the exam in the subject of prevention, results of the OSCE) will be combined and statistically analyzed in a database set up for this purpose. statistically. Only anonymized and accumulated data will be published. The data are protected from unauthorized access, as all data are stored on a password-protected computer at the Institute of General Practice in Würzburg. The interest of the evaluations is directed exclusively to group comparisons and not to individual analyses. The results will be published in the dissertation. To enable a pre-post comparison of the individual groups, we ask you to generate an individual code following our instructions and enter it in the field provided on the questionnaires. This code, which was developed in cooperation with the data protection officer, ensures that your data is collected and analyzed completely anonymously.

2. Individual code and randomized group

2.1 Individual code

- First and last letter of your maternal grandmother's first name (e.g. "UA" for UrsulA)
- First and last letter of your maternal grandfather's first name (e.g. "MN" for MartiN)
- Day of your mother's birthday (e.g. "03" for 3.October)

Please use large letters in block capitals
If you do not know a grandparent by name, please enter "XX" in the appropriate place.
__ __ / __ __ / __ __

2.2 I will participate in: □ seminar (cohort 1) □ online course (cohort 2)
The randomization has already been made, you can find your allocation on wuecampus

Attachment 1 to Ruck J, Tiedemann E, Sudmann J, Kübler A, Simmenroth A. *Evaluating the longitudinal effectiveness of a 5A model-based smoking cessation counselling course for medical students in family medicine placement.* GMS J Med Educ. 2025;42(1):Doc10. DOI: 103205/zma001734

3. Sociodemographic data and smoking status

3.1 gender: □ male □ female □ others

3.2 age (in years): ___

3.3 smoking status: □ non-smoker □ ex-smoker □ smoker □ no statement
(Explanation: non-smoker here means <100 cigarettes in a lifetime)

If you are a current smoker (non-smokers and ex-smokers please go to question 4.1)

3.4 I think the course will influence my smoking behaviour
Do not agree at all □ □ □ □ □ □ fully agree

3.5 I plan to quit smoking within the next six months
□ yes, with a concrete plan and date □ yes, I will try □ Let's see, in principle open to it □ no

4. Your personal attitude to the theme tobacco smoking

Now we are interested in **your** opinion. There are no right or wrong answers.

4.1 I am in general tolerant towards smokers.
Do not agree at all □ □ □ □ □ □ fully agree

4.2 I see smoking as an addictive disease
Do not agree at all □ □ □ □ □ □ fully agree

4.3 I am convinced that a physician can effectively influence the smoking behaviour of his/her patients.
Do not agree at all □ □ □ □ □ □ fully agree

4.4 I believe that patients feel "attacked" when medical personnel bring up the subject of smoking without being asked.
Do not agree at all □ □ □ □ □ □ fully agree

4.5 I think it is unnecessary for a physician to discuss the subject of smoking with every patient as patients know that smoking is harmful.
Do not agree at all □ □ □ □ □ □ fully agree

4.6 For consistent smoking counselling, it is an obstacle if the physician smokes himself/herself.
Do not agree at all □ □ □ □ □ □ fully agree

4.7 If I have the opinion to choose seminar or online course on my own, I would participate in:
□ online course □ I don’t care □ seminar

4.8 If you strongly prefer a teaching method on this subject, briefly tell us why: ____________

5. Self- assessment of your current level of knowledge about smoking

5.1 I am sufficiently informed about the risks of tobacco smoking.
Do not agree at all □ □ □ □ □ □ fully agree

5.2 I can explain to a patient the mechanisms of nicotine dependence in a comprehensible way.
Do not agree at all □ □ □ □ □ □ fully agree

5.3 I have already heard of the "5-A strategy" as a model of medical counselling.
Do not agree at all □ □ □ □ □ □ fully agree

5.4 I know what five steps and actions the 5 As concretely stand for.
Do not agree at all □ □ □ □ □ □ fully agree

5.5 Unless there is no contraindication, every smoking patient should be offered drug therapy during the counseling session.
Do not agree at all □ □ □ □ □ □ fully agree

5.6 I am aware of the following treatment options for nicotine cessation (check if aware):
□ Behavioral therapy □ Bupropion □ Varenicilin □ Nicotine replacement therapy

5.7 I feel safe to raise the topic of smoking with patients on my own even if the reason for the reason for consultation is not about smoking
Do not agree at all □ □ □ □ □ □ fully agree

5.8 I can confidently and independently take a smoking history
Do not agree at all □ □ □ □ □ □ fully agree

5.9 I can conduct a brief consultation with a smoking patient.
Do not agree at all □ □ □ □ □ □ fully agree

6. Experience in handling the subject of smoking during studies and previous clinical assignments

6.1 I have experienced that nursing staff or physicians with the subject of smoking on their own initiative.
Do not agree at all □ □ □ □ □ □ fully agree

6.2 I have experienced that nursing staff or physicians talk to a brief counselling session with a patient who smokes.
Do not agree at all □ □ □ □ □ □ fully agree

6.3 Smoking was discussed in detail in the following subjects (check if statement is true):
□ Psychology (preclinical) □ Pathology □ Environmental Medicine □Other: _______________

6.4 The topic of smoking is overrepresented in the course of study.
Do not agree at all □ □ □ □ □ □ fully agree

Supplemental material 2: Online self-assessment questionnaire to the 10th semester (winter semester 2020/21)

1. General information on the study "Evaluation of a longitudinal extension of a course on smoking cessation counselling in medical school" Part 2.

1. Why is this study being conducted and why should I participate?
By participating in the study, you will support the further development of teaching and help your fellow student Jessica Sudmann with her dissertation. The study is conducted by the Institute of General Medicine of the UKW. It is being conducted by Prof. Dr. Anne Simmenroth, Elena Tiedemann and Jessica Sudmann (as a doctoral student) conducted. Participation in the study is voluntary.

2. What is the procedure of the study and what should be considered?
In the cross-sectional field of prevention, a course on nicotine consumption already took place in the winter semester 2018/19, in the Summer Semester 2020 as part of the seminar "Psychosomatic/Addiction", the short-intervention according to the 5 As was refreshed. Now in the winter semester 2020/2021 in the placement in General Medicine, the practical implementation of what has been learned will be examined. The survey that now follows is intended to assess the feasibility of and experiences with nicotine cessation counselling.

3. What is the aim of the study?
The aim of the study is to investigate how much knowledge on the topic of "tobacco use" from the winter semester 2018/2019 can be reproduced in the cross-sectional field of prevention. In addition, the implementation of conducting a counselling in the placement in General Medicine will be investigated.

4. What happens with your data?
The data will be collected in pseudonymous form, i.e. coded without revealing identity, name or date of birth. Your data will be stored electronically on password-protected data carriers of the Institute of General Medicine of the UKW. Only staff members of the study have access to the data. If you withdraw your participation in the study, the data will be deleted. The results of the study will be published in the dissertation, whereby your identity will of course remain anonymous.

5. Who can I contact if I have further questions?
Jessica Sudmann (jessica.sudmann@stud-mail.uni-wuerzburg.de)

2. Sociodemographic data and smoker status

2.1 Individual code

- First and last letter of your maternal grandmother's first name (e.g. "UA" for UrsulA)
- First and last letter of your maternal grandfather's first name (e.g. "MN" for MartiN)
- Day of your mother's birthday (e.g. "03" for 3.October)

Please use large letters in block capitals
If you do not know a grandparent by name, please enter "XX" in the appropriate place.
__ __ / __ __ / __ __

IMPORTANT! Please be sure to use the same code as in the smoking cessation study in the prevention in the winter semester 18/19 or in general medicine in the summer semester Summer semester 20, if you participated in it!

2.2 gender □ male □ female □ others

2.3 age (in years) ___

2.4 I have already completed apprenticeship in the medical field □ yes □ no

2.5 current smoking status: □ non-smoker □ ex-smoker □ smoker □ no statement
(Explanation: non-smoker here means <100 cigarettes in a lifetime)

2.6 smoker □ sometimes (e.g. paties) □ regularly (e.g. several times per week)

2.7 I have participated in the following courses (multiple selection)
□ tobacco consumption course in the cross-sectional field prevention (6th semester)
□ Refresher course on the 5 As in the course Psychosomatic/Addiction in the subject General Medicine
□ I had no course on the topic of tobacco consumption (e.g. because of several semesters off, pregnancy, stays abroad or similar)
□ I do not remember.
□ Other (please specify in free text field)

2.8 tobacco consumption course in the cross-sectional field prevention (6th semester)
□ online □ face-to-face □ I do not remember

3. Feasibility of a smoking cessation counselling in the placement of General Medicine

3.1 As part of the placement, I conducted a nicotine cessation counselling. □ yes □ no

3.2 Please give us reasons why you were unable to complete a smoking cessation counselling.
(free text field)

3.3 I assess the time required for a short intervention by the concept of the 5 As, e.g. in an anamnesis, as feasible.
Do not agree at all □ □ □ □ □ □ fully agree

3.4 I have performed the short intervention for nicotine cessation counselling in the context of...
□ Health check-up (GU) □ Anamnesis
□ DMP COPD/ Asthma
□ Patient came with an infection of the respiratory tract
□ Other (please specify in free text please specify)

3.5 I have performed more than one short intervention on nicotine cessation counselling.
□ yes □ no

3.6 How much time did it take?
□ Less than 5 Minutes
□ Between 5 and 10 minutes
□ More than 10 Minutes

3.7 The teaching physician helped me with the selection of an appropriate patient.
Do not agree at all □ □ □ □ □ □ fully agree

3.8 smoking cessation counselling materials and support programs... (multiple choice).
□ ... are sufficiently known to me.
□ ... I have used during my nicotine cessation counselling
□ ... were available for patients in the teaching general practice (e.g. in the waiting room)

4. Experiences, Satisfaction, feeling of confidence and self-assessment of communication competences

If you have had several smoking cessation counselling, evaluate one conversation of your choice.
4.1 I conducted the nicotine cessation counselling according to the principle of the 5 As.
□ Yes □ No □ Partly

4.2 The patient responded cooperatively to the approach.
Do not agree at all □ □ □ □ □ □ fully agree

4.3 The patient was at the following motivation level.
□ Ready □ Unsure □ Not ready

4.4 I have used the following As: (Tick as appropriate and assess safety in the following)
□ Ask □ Advise □ Assess □ Assist □ Arrange

4.5 I felt confident in the use of the "Ask" item.
Do not agree at all □ □ □ □ □ □ fully agree
4.6 I felt confident in the use of the "Advise" item.
Do not agree at all □ □ □ □ □ □ fully agree

4.7 I felt confident in the use of the "Assess" item.
Do not agree at all □ □ □ □ □ □ fully agree

4.8 I felt confident in the use of the "Assist" item.
Do not agree at all □ □ □ □ □ □ fully agree

4.9 I felt confident in the use of the “Arrange" item.
Do not agree at all □ □ □ □ □ □ fully agree

4.10 Overall, I felt confident in conducting the brief intervention based on the 5A model.
Do not agree at all □ □ □ □ □ □ fully agree

4.11 I am satisfied with the performance of my nicotine cessation counselling.
Do not agree at all □ □ □ □ □ □ fully agree

4.12 I feel safe to raise the topic of smoking with patients on my own even if the reason for the reason for consultation is not about smoking
Do not agree at all □ □ □ □ □ □ fully agree

4.13 Through the placement my skills in nicotine cessation counselling have been
decreased □ □ □ □ □ □ increased

5. Motivation and individual attitude to the topic smoking cessation counselling

5.1 I am convinced that a physician can effectively influence the smoking behaviour of his/her patients.
Do not agree at all □ □ □ □ □ □ fully agree

5.2 I believe that patients feel "attacked" when medical personnel bring up the subject of smoking without being asked.
Do not agree at all □ □ □ □ □ □ fully agree

5.3 I think it is important to counsel patients about their smoking behaviour as a medical student, intern or physician.
Do not agree at all □ □ □ □ □ □ fully agree

5.4 I think, I’m integrating the smoking counselling in my future work.
Do not agree at all □ □ □ □ □ □ fully agree

5.5 I feel well prepared to conduct smoker counselling sessions independently as a physician.
Do not agree at all □ □ □ □ □ □ fully agree

6. Wishes and ideas?

6.1 What school grade do you give the entire course "Nicotine Cessation” incl. all components (seminar in prevention, refresher course in general in general medicine and implementation in the placement)
□ 1 □ 2 □ 3 □ 4 □ 5 □ 6

6.2 Note here wishes, ideas, positive and negative feedback
(free text field)

Supplemental material 3: Items of variables “attitude” and “confidence”

		Items of questionnaires	6th	10th
**Attitude**	**1**	**I am convinced that a physician can effectively influence the smoking behaviour of his patients.**	**X**	**X**
**Attitude**	**2**	**I believe that patients feel "attacked" when medical personnel bring up the subject of smoking without being asked.**	**X**	**X**
**Attitude**	**3**	I think it is important to counsel patients about their smoking behaviour as a medical student, intern or physician.		X
**Attitude**	**4**	I think, I’m integrating the smoking cessation counselling in my future work.		
**Confidence**	**1**	**I feel confident to raise the issue of smoking with patients on my own initiative, even if the reason for consultation is not about smoking.**	**X**	**X**
**Confidence**	**2**	I felt confident in the implementation of the brief intervention according to the concept of the 5As.		X
**Confidence**	**3**	I am satisfied with the conduct of my smoking cessation counselling.		X
**Confidence**	**4**	I feel well prepared to conduct smoker counselling sessions independently as a physician.		X
**Competences**		My competences in smoking cessation counselling changed afterwards (5-point-likert-scale: 1=decreased, 5=increased).		X
